# Cyberchondria in Older Adults and Its Relationship With Cognitive Fusion, Health-Related Quality of Life, and Mental Well-Being: Mediation Analysis

**DOI:** 10.2196/70302

**Published:** 2025-05-21

**Authors:** Richard Huan Xu, Vladan Starcevic

**Affiliations:** 1 Department of Rehabilitation Sciences Faculty of Health and Social Sciences Hong Kong Polytechnic University Kowloon China (Hong Kong); 2 Faculty of Medicine and Health Sydney Medical School, Nepean Clinical School, Brain and Mind Centre University of Sydney Sydney Australia

**Keywords:** cyberchondria, cognitive fusion, health-related quality of life, well-being, mediation

## Abstract

**Background:**

Cyberchondria is the compulsive searching for health information online that continues despite harmful effects. It leads to increased health anxiety and lower health-related quality of life (HRQOL). Older adults face higher risks of cyberchondria due to their limited digital literacy skills and more frequent health concerns. However, researchers have not thoroughly studied how cyberchondria affects this age group.

**Objective:**

This study aimed to explore cyberchondria in the older population and investigate its relationship with cognitive fusion (ie, the tendency to become entangled with thoughts and perceive them as literal truths that dictate behavior), HRQOL, and mental well-being.

**Methods:**

A web-based, cross-sectional survey was conducted in May 2024 with a sample of 638 participants from China aged ≥60 years recruited through the online panel of a survey company. The participants completed questionnaires assessing cyberchondria (using the Cyberchondria Severity Scale-12 [CSS-12]), cognitive fusion, HRQOL, and mental well-being. Structural equation modeling (SEM) was used to assess the hypothesized mediation model, and standardized estimates and their 95% CIs were calculated for all structural paths.

**Results:**

Participants had a mean CSS-12 score of 40 (SD 8.5), suggesting a fairly high level of cyberchondria in this sample. Participants with a higher socioeconomic status tended to report lower levels of cyberchondria. The SEM showed that cyberchondria was positively associated with cognitive fusion (β=0.505, *P*<.001 for both models) and negatively associated with HRQOL (β=–0.221, *P*<.001) and mental well-being (β=–0.212, *P*<.001). The mediation model showed a good fit and demonstrated that cognitive fusion fully mediated the total effect of cyberchondria on HRQOL and mental well-being.

**Conclusions:**

Cyberchondria may be more prominent in older Chinese adults, especially those residing in rural areas and with a lower socioeconomic status. Additionally, cyberchondria can enhance cognitive fusion, contributing to poor HRQOL and mental well-being. Interventions focused on “defusing” cyberchondria-relevant thoughts may help reduce maladaptive behaviors associated with cyberchondria and improve the overall well-being of older populations.

## Introduction

### Background

The internet can amplify health anxiety by creating an environment in which distinguishing credible online health information from that derived from unreliable sources may be difficult, often leading to confusion and fear [1,2]. Cyberchondria refers to excessive or repeated online health information seeking that persists despite negative consequences and is associated with increased health anxiety [3] and decreased health-related quality of life (HRQOL) [4,5], with its toll including diminished well-being and compromised daily functioning. Cyberchondria and health anxiety are closely intertwined, and studying this link is crucial to address the growing impact of cyberchondria. The rise of cyberchondria is intimately linked to the digital revolution, and online misinformation and worst-case scenarios often lead to catastrophic interpretations that exacerbate anxiety. The internet also facilitates confirmation bias, allowing individuals to seek information that aligns with their existing fears, reinforcing anxiety. Easy access to information facilitates compulsive checking behaviors, which provide temporary relief but ultimately perpetuate anxiety. This creates a vicious cycle, in which increased anxiety leads to more compulsive online health information seeking [4,6,7].

### Cyberchondria in Older Adults

Older adults face heightened vulnerability to cyberchondria. Their vulnerability stems not only from lower digital literacy and more frequent health concerns [8,9] but also from their generation’s unique beliefs and life experiences [10]. Older adults, particularly those from generations that emphasized deference to authority figures, may uncritically accept health-related information presented in an authoritative tone, even from unreliable online sources [11]. This attitude, coupled with passive health information-seeking habits developed in an era of limited access to medical knowledge, heightens susceptibility to misinformation and catastrophic health interpretations.

Moreover, older age brings an expectation of decline, a psychological state shaped by imminent health risks, accumulated losses, and direct experiences with chronic illness [12]. These experiences may foster hypervigilance toward bodily changes, prompting compulsive online searches that reinforce anxiety through confirmation bias. The interplay of factors, such as cohort beliefs, anticipatory health anxiety, and cumulative adversity, may create a unique pathway for cyberchondria in older adults, distinct from younger populations. Despite these risks, limited research has focused on cyberchondria in this age group, even as studies highlight their frequent use of the internet for health information, which may increase their anxiety and distress [12-14].

### HRQOL and Cyberchondria

HRQOL is a multidimensional construct that encompasses an individual’s physical health, mental health, social functioning, and role functioning [15]. HRQOL is particularly affected by cyberchondria, as the anxiety and stress from constant health-related searches have a negative impact on emotional well-being. This anxiety can also manifest as psychosomatic symptoms, affecting physical health and daily functioning.

Unravelling the mechanisms linking cyberchondria to HRQOL is essential, as individual differences in coping strategies, digital literacy, and pre-existing health conditions interact dynamically with the aforementioned specific vulnerabilities in older adults. As cyberchondria becomes more prevalent [16], targeted interventions must address not only the skill gaps but also the ingrained cognitive and emotional patterns that predispose older adults to its harms, ultimately safeguarding their well-being in an increasingly digital health landscape.

### Mental Well-Being and Cyberchondria

Mental well-being and HRQOL are distinct concepts that address different dimensions of human experience. Unlike HRQOL, which quantifies the impact of health on physical, social, and role functioning, mental well-being focuses on emotional and psychological states and encompasses positive aspects such as happiness, life satisfaction, resilience, and a sense of purpose [17]. Rooted in frameworks like positive psychology, mental well-being relates to the presence of flourishing mental states (eg, optimism, self-acceptance) and absence of psychological distress (eg, anxiety, depression). Cyberchondria can jeopardize mental well-being by heightening anxiety about one’s health [18]. It can also destabilize emotional health, trigger obsessive thought patterns, and impair resilience, ultimately eroding the core components of mental well-being and leaving individuals trapped in a cycle of distress and hypervigilance [19].

Elucidating the mechanisms connecting cyberchondria to mental well-being is vital, especially for older adults who face unique risks due to diminished digital literacy, heightened health concerns, and reduced adaptability to stress [20]. Factors such as pre-existing anxiety, limited ability to critically evaluate online content, and a propensity for catastrophic thinking can intensify cyberchondria’s impact, making older adults particularly susceptible to its psychological toll [21]. With the growing prevalence of digital health-seeking behaviors, research must prioritize these interactions to inform robust, tailored interventions, combining enhanced digital education with cognitive-behavioral strategies, to break the cycle of anxiety and safeguard mental well-being in this vulnerable population [22].

### Cognitive Fusion and Cyberchondria

Cognitive fusion refers to the tendency for behavior to be overly regulated and influenced by cognition. Cognitive fusion occurs when individuals get overly entangled with their thoughts, treating them as literal truths rather than just mental phenomena [23]. Although cognitive fusion is primarily a state phenomenon, some individuals may experience it more persistently, leading to patterns that resemble a trait. This means that certain people might be more prone to cognitive fusion across various situations, making it appear as a stable, trait-like characteristic [24]. Studies have shown that cognitive fusion is strongly associated with health care avoidance and weight stigma [25] and that higher levels of cognitive fusion are associated with increased anxiety and depression and poorer HRQOL [26,27]. Cognitive fusion is also related to rumination, shame, and reduced self-compassion [28]. Cognitive fusion involving anxious thoughts can exacerbate anxiety-related maladaptive behaviors, such as avoidance, checking, and reassurance seeking. The inability to distance oneself from such thoughts perpetuates the anxiety cycle and strengthens the perceived validity of the thoughts [29].

Previous studies have shown an association between cyberchondria and metacognitive beliefs [30,31], a concept potentially linked to cognitive fusion. Although metacognitive beliefs refer to ways of appraising one’s thoughts and other cognitive functions, cognitive fusion is about a strong attachment to one’s thoughts and their perceived truth. Compared with cognitive fusion, metacognitive beliefs reflect a broader construct, can be either positive or negative, and lack an element of attachment to one’s own thoughts, regardless of the nature of these thoughts. Both constructs influence how individuals experience and respond to their thoughts, but considering the potential role that cognitive fusion can play in psychopathology, it has not received sufficient attention from researchers in the context of cyberchondria.

Although cognitive fusion can affect people of all ages, older adults may be more severely impacted than their younger counterparts, given their thoughts about numerous life transitions, including retirement, loss of loved ones, high risks of social isolation, and declining health [32]. Cognitive fusion may amplify health-related anxiety of older adults by making it difficult for them to separate their concerning health-related thoughts from the reality about their health. However, limited empirical evidence exists on the relationship between cognitive fusion and health anxiety among older adults [33]. Studying cognitive fusion in this demographic may help develop tailored interventions to enhance coping mechanisms of older adults and improve their mental health and overall HRQOL [34].

The relationship between cognitive fusion and cyberchondria can be understood through the lens of cognitive processes and reinforcement. Cognitive fusion may contribute to negative perceptions of online health information [35]. In other words, the threatening thoughts about online health information, especially if this information is ambiguous or incongruent, are experienced as real and not a product of one’s own interpretation or perception. This increases anxiety and makes a person search compulsively for health information in an effort to alleviate the sense of threat, thus leading to cyberchondria [29,36]. However, such behavior only reinforces the sense of threat, especially in the long run. For older adults whose health-related searches are often driven by anticipatory anxiety [12], seeking ambiguous or alarming information may deepen cognitive fusion (“I must be ill because I keep reading about symptoms”), creating a feedback loop. This aligns with studies showing that repetitive behaviors amplify cognitive rigidity in aging populations [33]. Thus, we hypothesized cyberchondria as a behavioral trigger that intensifies cognitive fusion, which in turn erodes HRQOL.

### Research Questions and Hypotheses

#### Research Questions

Drawing from the theoretical and empirical considerations outlined earlier, this study proposed 2 research questions to guide the investigation. First, what are the levels of cyberchondria and cognitive fusion among older adults, and which sociodemographic factors correlate with these constructs? Second, how are cyberchondria, cognitive fusion, HRQOL, and mental well-being interrelated in older adults? To address the second question, 5 hypotheses were formulated. The conceptual framework is presented in Multimedia Appendix 1.

#### Hypothesis 1

Prior research has demonstrated that cyberchondria contributes to functional impairment and diminished quality of life [18]. In older adults, this effect may be amplified due to age-related vulnerabilities, such as reduced online health literacy and the presence of chronic illnesses, which heighten susceptibility to health anxiety and its consequences [37]. Therefore, we proposed, as hypothesis 1 (H1), that cyberchondria negatively impacts HRQOL in older adults.

#### Hypothesis 2

Studies have shown that excessive online health information seeking is linked to increased psychological distress, including heightened anxiety and reduced emotional well-being [38,39]. Older adults may be particularly vulnerable to these effects due to declining physical health, reduced resilience to stress, and limited digital literacy, which can exacerbate the psychological toll of cyberchondria. Thus, we hypothesized, as hypothesis 2 (H2), that cyberchondria negatively impacts mental well-being in older adults.

#### Hypothesis 3

Research has indicated that cognitive fusion, characterized by entanglement with thoughts and treating them as literal truths, is associated with anxiety-driven behaviors [40]. Cyberchondria, as a behavioral pattern driven by health anxiety, may similarly foster cognitive fusion by reinforcing rigid and catastrophic thought patterns about health. Accordingly, we proposed, as hypothesis 3 (H3), that cyberchondria positively impacts cognitive fusion in older adults.

#### Hypothesis 4

The theoretical framework of acceptance and commitment therapy, supported by studies, suggests that cognitive fusion amplifies the negative effects of maladaptive thoughts on well-being, including health-related outcomes [41]. In the context of cyberchondria, older adults who excessively seek online health information may develop rigid, catastrophic cognitions that impair HRQOL through cognitive fusion [42]. Therefore, we hypothesized, as hypothesis 4 (H4), that cognitive fusion mediates the negative impact of cyberchondria on HRQOL in older adults.

#### Hypothesis 5

Evidence from prior research indicates that cognitive fusion intensifies the emotional impact of anxiety-driven behaviors, mediating the association between repetitive thought patterns and psychological distress [40]. For older adults, cyberchondria may similarly exacerbate emotional distress through cognitive fusion, undermining mental well-being [23]. Thus, we proposed, as hypothesis 5 (H5), that cognitive fusion mediates the negative impact of cyberchondria on mental well-being in older adults.

## Methods

### Data and Participants

This study used data from a web-based cross-sectional survey conducted between April 2024 and May 2024 to examine the health and social status of China’s older population. The participants were recruited through Wenjuanxing, a Chinese survey company with an online panel of over 2.6 million members. The inclusion criteria were as follows: (1) age ≥60 years, (2) ability to read and speak Mandarin, (3) absence of cognitive impairments, and (4) ability to provide informed consent. Eligible participants were invited to complete a series of questionnaires starting with an informed consent form. Only those who consented to participate then completed the questionnaire. Several measures were implemented to ensure data quality. First, we used CAPTCHA (Completely Automated Public Turing test to tell Computers and Humans Apart) to prevent bot submissions, ensuring that only genuine human respondents participated. We also conducted a time analysis to exclude implausibly rapid responses, which could indicate a lack of thoroughness. Additionally, we limited submissions to one per IP address within a set time frame to prevent duplicate entries. To further enhance data integrity, we identified and filtered parallel response patterns, including both consistent and repetitive patterns, which could suggest inattentive or automated responses.

### Measures

The standard and validated Chinese versions of all instruments were administered. The English versions are included in Multimedia Appendix 2 for reference only.

#### Cyberchondria

The severity of cyberchondria was measured using the Cyberchondria Severity Scale-12 (CSS-12). It possesses good psychometric properties, comparable to those of the original version, and has been validated in the Chinese population [43]. The CSS-12 items were rated on a Likert-type scale ranging from 1 (never) to 5 (always). The total score ranges from 12 to 60, with higher scores indicating higher levels of cyberchondria.

#### Cognitive Fusion

The Cognitive Fusion Questionnaire (CFQ) was designed to measure the extent to which individuals are entangled in their thoughts [23]. It consists of 7 items, each rated on a 7-point Likert scale ranging from 1 (never true) to 7 (always true). Total scores range from 7 to 49, with higher scores reflecting a higher level of cognitive fusion. The psychometric properties of the CFQ have been reported to be satisfactory in the Chinese population [44].

#### HRQOL

The EQ-5D-5L was used to measure HRQOL in this study. It comprises 5 health-related items (mobility, self-care, usual activities, pain or discomfort, and anxiety or depression), each rated on a 5-point Likert scale ranging from 1 (no problems) to 5 (extreme problems) [45]. To reflect HRQOL, all health states described by the descriptive system can be converted into a single utility score using a scoring algorithm based on public preferences. This study used the EQ-5D-5L China value set and scoring algorithm [46].

#### Mental Well-Being

The World Health Organization-5 Well-Being Index (WHO-5) is a widely recognized and validated tool for assessing subjective psychological well-being [47]. It consists of 5 questions that measure positive mood, vitality, and general interest over the past 2 weeks. Each item is rated on a 6-point Likert scale ranging from 0 (not present) to 5 (constantly present), allowing for a maximum score of 25. Higher scores indicate better well-being. The psychometric properties of the WHO-5 in the Chinese population have been reported to be satisfactory [48].

### Statistical Analysis

Descriptive statistics were used to analyze participants’ sociodemographic characteristics. Continuous variables (eg, age) were analyzed using means and SDs, whereas categorical variables (eg, sex) were analyzed using frequencies and percentages. Pearson correlation coefficients (*r*) were calculated to determine associations between variables, with *r* values ≥0.3 and ≥0.5 indicating moderate and large effects, respectively [49]. ANOVA was conducted to assess the differences in CSS-12 scores across different socioeconomic groups.

We used structural equation modeling (SEM) with full-information likelihood estimation to assess the hypothesized mediation model. Latent variables for cyberchondria (CSS-12), HRQOL (EQ-5D-5L), mental well-being (WHO-5), and cognitive fusion (CFQ) were created based on the sum score of the 4 instruments. Respondents’ characteristics (ie, sex, age, education level, and presence of a chronic condition) were included in the model for adjustment. To assess the direct, indirect, and total effects, we used 5000 bootstrapped samples, derived effect estimates, and bias-corrected 95% CIs [50]. We assessed model fit using a Tucker-Lewis index>0.90, comparative fit index>0.90, root mean square error of approximation<0.05, and standardized root mean square residual<0.05 [51]. We set significance at *P*<.05 (2-tailed) and conducted all statistical analyses using R software [52].

### Ethical Considerations

This study was performed in line with the principles of the Declaration of Helsinki. The study protocol and informed consent were approved by the Institutional Review Board of Hong Kong Polytechnic University (reference ID: HSEARS20230502006). Informed consent was obtained from all the participants. All data were anonymized to protect participants’ privacy and ensure confidentiality.

## Results

### Participants’ Sociodemographic Characteristics

Table 1 presents the sociodemographic characteristics of the 638 survey participants. The majority were men, comprising 64.1% (409/637) of the sample. In terms of educational background, 51.4% (328/637) had a high school education or less, 25.9% (165/637) had attended college, and 22.7% (145/637) held a university degree. Regarding their household registration, 37.5% (239/637) were from rural areas, while 62.5% (399/637) were from urban areas. When asked about their perceived socioeconomic status, 11% (70/637) considered themselves below the local average, 79.1% (505/637) felt they were equal to it, and 9.9% (63/637) viewed themselves as above it. Moreover, 60.7% (387/637) were long-term caregivers, and 55.3% (353/537) had chronic conditions.

**Table 1:** Participants’ demographics and socioeconomic status (n=637).

Characteristics		Results, n (%)
**Sex**		
	Male	409 (64.1)
	Female	229 (35.9)
**Age (years)**		
	60-65	533 (81.6)
	66-79	105 (18.4)
**Educational level**		
	High school or less	328 (51.4)
	College	165 (25.9)
	University	145 (22.7)
**Household registration**		
	Rural	239 (37.5)
	Urban	399 (62.5)
**Marital status**		
	Single	9 (1.4)
	Married	578 (90.6)
	Divorced/widow(er)	51 (8)
**Perceived socioeconomic status**		
	Lower than local average	70 (11)
	Equal to local average	505 (79.1)
	Higher than local average	63 (9.9)
**Long-term caregiver**		
	Yes	387 (60.7)
	No	251 (39.3)
**Chronic conditions**		
	Yes	353 (55.3)
	No	285 (44.7)

### Profiles of Cyberchondria, HRQOL, Mental Well-Being, and Cognitive Fusion and Associations Between Them

Table 2 presents the participants’ scores on the 4 assessment instruments, while Table 3 shows the correlations among the instruments. The average CSS-12 (cyberchondria) score was 40 (SD 8.5) points. The mean WHO-5 (mental well-being) score was 20.4 (SD 5.0) points, the mean EQ-5D-5L (HRQOL) score was 0.8 (SD 0.19) points, and the mean CFQ (cognitive fusion) score was 36.8 (SD 12.3) points. The Cronbach α coefficients for all 4 instruments were above 0.8, indicating good internal consistency and reliability.

**Table 2:** Measure profiles.

Measures	Mean (SD)	Median (range)	Cronbach α
CSS-12^a^	40 (8.5)	42 (12-58)	0.87
WHO-5^b^	20.4 (5.0)	21 (5-30)	0.87
EQ-5D-5L	0.8 (0.19)	0.88 (0.08-1)	0.83
CFQ^c^	36.8 (12.3_	39 (9-63)	0.94

^a^CSS-12: Cyberchondria Severity Scale-12.

^b^WHO-5: World Health Organization-5 Well-Being Index.

^c^CFQ: Cognitive Fusion Questionnaire.

**Table 3:** Correlations between the measures.

Measures			CSS-12^a^		WHO-5^b^		EQ-5D-5L		CFQ^c^
**CSS-12**									
	*r*	—^d^		–0.20		–0.30		0.50	
	*P* value	—		<.001		<.001		<.001	
**WHO-5**									
	*r*	–0.20		—		0.36		–0.42	
	*P* value	<.001		—		<.001		<.001	
**EQ-5D-5L**									
	*r*	–0.30		0.36		—		–0.48	
	*P* value	<.001		<.001		—		<.001	
**CFQ**									
	*r*	0.50		–0.42		–0.48		—	
	*P* value	<.001		<.001		<.001		—	

^a^CSS-12: Cyberchondria Severity Scale-12.

^b^WHO-5: World Health Organization-5 Well-Being Index.

^c^CFQ: Cognitive Fusion Questionnaire.

^d^Not applicable.

Mental well-being (WHO-5) was positively correlated with HRQOL (EQ-5D-5L; *r*= 0.36, *P*<.001), suggesting that greater well-being aligns with better HRQOL. Conversely, mental well-being was negatively correlated with cognitive fusion (CFQ; *r*=–0.42, *P*<.001), indicating that higher well-being corresponds with reduced cognitive fusion. Similarly, HRQOL was negatively correlated with cognitive fusion (*r*=–0.48, *P*<.001), demonstrating that better HRQOL is linked to lower cognitive fusion. Cyberchondria (CSS-12) had negative associations with mental well-being (*r*=–0.2, *P*<.001) and HRQOL (*r*=–0.3, *P*<.001) and a positive association with cognitive fusion (*r*=0.5, *P*<.001). These results suggest that elevated cyberchondria is associated with poorer mental well-being, diminished HRQOL, and heightened cognitive fusion (Table 3).

### Differences in Cyberchondria Across Sociodemographic Groups

Table 4 illustrates the differences in cyberchondria (CSS-12) scores across various sociodemographic groups. Although no statistically significant differences were observed between the sexes, substantial variations were evident in all other group comparisons. Notably, individuals with higher educational levels, who resided in urban areas, who were younger, who had a higher socioeconomic status, who did not need caregivers, and who had no chronic conditions had lower cyberchondria scores.

**Table 4:** Participants’ responses on the Cyberchondria Severity Scale-12 (CSS-12) and Cognitive Fusion Questionnaire (CFQ), stratified by demographics.

Demographic characteristics			Cyberchondria (CSS-12)					Cognitive fusion (CFQ)		
			Mean (SD)		*P* value		Mean (SD)			*P* value
**Sex**					.36					.72
	Male	39.8 (8.8)				37.0 (12.4)				
	Female	40.4 (7.8)				36.6 (12.2)				
**Educational level**					<.001					<.001
	High school	41.0 (7.8)				38.5 (12.2)				
	College	39.5 (8.3)				35.8 (11.8)				
	University	38.3 (9.8)				34.5 (12.7)				
**Household registration**					<.001					<.001
	Rural	42.6 (6.4)				41.6 (10.0)				
	Urban	38.5 (9.2)				34.0 (12.7)				
**Age group (years)**					.02					.02
	60-65	38.3(9.3)				34.4(12.5)				
	66-79	40.3(8.2)				37.4(12.2)				
**Socioeconomic status**					.01					<.001
	Lower than average	42.1 (8.7)				43.4 (11.0)				
	Average	39.9 (8.2)				36.3 (12.1)				
	Higher than average	38.5 (9.6)				34.5 (13.6)				
**Do you need a caregiver?**					.001					<.001
	Yes	39.1 (8.9)				35.6 (12.8)				
	No	41.4 (7.5)				38.9 (11.3)				
**Do you have chronic condition?**					<.001					<.001
	Yes	41.1 (7.7)				40.0 (11.0)				
	No	38.6 (9.1)				33.0 (12.7)				

### Differences in Cognitive Fusion Across Sociodemographic Groups

The differences in respondents’ cognitive fusion across socioeconomic groups were similar to those found for levels of cyberchondria. Participants exhibited significantly higher cognitive fusion if they had lower educational levels, lived in rural areas, were older, had a lower socioeconomic status, and had caregivers or chronic conditions (Table 4).

### Results of the Hypothesized Mediation Model

**Figure 1:**
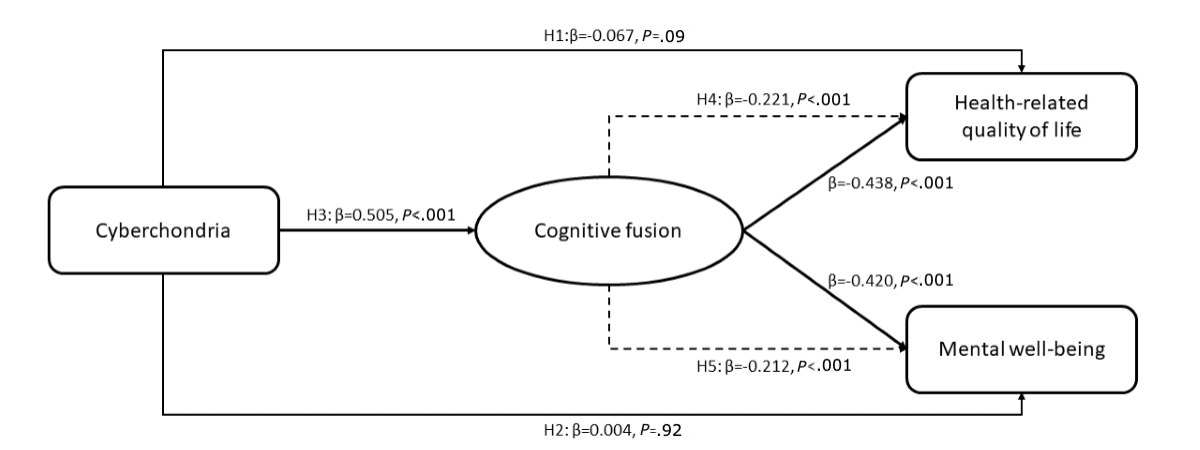
Results of mediation analysis.

Figure 1 shows the results of the SEM analysis. The mediation model, adjusted for sex, age, and education level, demonstrated acceptable model indices (comparative fit index=0.997, Tucker-Lewis index=0.993, root mean square error of approximation=0.017), suggesting that unmeasured potential confounders were relatively unlikely to affect the results. Outcomes of the SEM analysis showed the lack of statistically significant direct effects between cyberchondria and HRQOL (β=–0.067, *P*=.09; H1 is not supported) and between cyberchondria and mental well-being (β=0.004, *P*=.92; H2 is not supported). The coefficients of the mediation paths were statistically significant. Cyberchondria was significantly positively associated with cognitive fusion (β=0.505, *P*<.001; H3 is supported) with significant negative indirect associations with HRQOL (β=–0.221, *P*<.001; H4 is supported) and mental well-being (β=–0.212, *P*<.001; H5 is supported).

## Discussion

### Principal Findings

This study demonstrates that the levels of cyberchondria in the sample of older Chinese adults were rather high. Our participants had an average score of 40 (of the maximum score of 60) on the CSS-12, which surpassed the mean scores found in earlier research [53-56]. According to the CSS-12 cutoff value established by Xu [57], 60% of our participants exhibited significant features of cyberchondria. These results provide empirical evidence that internet-related mental health issues affect older adults, challenging the belief that such problems are mainly of relevance for younger individuals [58,59]. Additionally, the mediation analysis revealed that associations between cyberchondria and both HRQOL and mental well-being were complex, as no significant direct effects were observed in the model (H1 and H2 were not supported). However, cyberchondria appeared to have a negative and indirect effect on HRQOL and mental well-being by enhancing cognitive fusion among older adults (H3, H4, and H5 were supported). This highlights the importance of addressing cyberchondria not just as a mental health issue but also as a broader HRQOL concern. Interventions that aim to improve overall well-being, not just reduce anxiety, in this population should be encouraged. However, since the significant associations disappeared in the mediation models, the mechanisms relating cyberchondria to HRQOL and mental well-being might be complex and call for further research scrutiny.

The factors contributing to the high levels of cyberchondria are complex. Our study found that older adults with a lower socioeconomic status reported significantly higher cyberchondria levels than those with a higher socioeconomic status. This finding is consistent with results of previous studies conducted in other populations. For example, a Turkish study revealed a significant relationship between cyberchondria levels and family income among adolescents [60]. Several reasons may help explain this. Older individuals with a lower socioeconomic status often have limited access to health care resources, poor internet connectivity, and lower eHealth literacy [61,62]. These factors may compel them to rely heavily on potentially unreliable online health information. Moreover, older individuals with a lower socioeconomic status are more likely to experience social isolation and have smaller support networks [63,64], which may exacerbate their health anxiety and lead to higher levels of cyberchondria. Given these complexities, cyberchondria should be studied within the broader context of psychosocial determinants of health rather than as a simple health-related phenomenon.

A significant difference in CSS-12 scores emerged between urban and rural residents. Older urban residents exhibited lower levels of cyberchondria than their rural counterparts. Although a previous study found that health anxiety is more prevalent and severe in rural areas than in urban areas [65], the study empirically confirmed that the variation in cyberchondria levels was linked to household registration. The reasons for the striking difference in cyberchondria levels between urban and rural older adults largely overlap with the aforementioned reasons for the difference in cyberchondria levels between older individuals with higher and lower socioeconomic statuses. In addition, the urban migration of younger generations in China often leaves older family members in rural areas, which can hinder older people’s access to direct support for using digital technologies [66]. Thus, a shift to digital health information, emphasized by the government, may become a challenge for rural residents [67,68].

The cognitive fusion scores in our sample of older adults were slightly higher than those reported in younger populations [32,69] but lower than those in populations with mental health problems, such as depression [70] and suicidal intention [71]. Gillanders et al [26] found that cognitive fusion is negatively associated with HRQOL, which is consistent with our findings. Additionally, we found a significant association between cognitive fusion and education; older respondents with higher educational levels had lower levels of cognitive fusion. Previous studies indicated that education often enhances cognitive flexibility [72], improves critical-thinking skills [73], and boosts problem-solving abilities [74]. These factors may help better-educated individuals avoid rigid thought patterns, thus reducing their proneness to cognitive fusion.

The mediation model revealed that cyberchondria may increase cognitive fusion, leading individuals to become overly entangled with their thoughts about online health information. Heightened cognitive fusion can negatively impact HRQOL and mental well-being by making it challenging for older individuals to manage their distressing thoughts and emotions. This finding provides support to H2 of this study and partially aligns with the findings of previous studies showing that cognitive fusion significantly mediates the relationship between individual well-being and other health-related factors [75-78].

Building on previous research and clinical findings, our study extends the understanding of how cognitive fusion may serve as a potential mechanism by which cyberchondria causes negative health outcomes [79]. These findings provide valuable insights into how excessive online health information seeking may affect mental health and other functions. This study has clinical implications, as it suggests that interventions targeting cognitive fusion may be effective in mitigating the impact of cyberchondria on HRQOL and mental well-being.

As populations worldwide age, many countries are experiencing a surge in internet use among older adults, driven by increased access to smartphones and online platforms. The rising trend of cyberchondria among Chinese older adults found in this study is very relevant for other regions with aging populations, such as Japan, the United States, and much of Europe, where older adults increasingly turn to the web for health information [80]. The internet’s role in amplifying anxiety through misinformation and compulsive online behavior is a universal concern [81]. Other countries can learn from China’s experience to prepare for similar challenges, particularly where health care systems might struggle because of the increasing number of older patients with high levels of health anxiety and cyberchondria.

### Strengths and Limitations

This is the first study conducted in China to specifically examine cyberchondria and its sociodemographic correlates in the older population. The novelty of this study lies in its examination of previously overlooked relationships between cyberchondria, cognitive fusion, HRQOL, and mental well-being in this population. The findings of this study enhance our understanding of cyberchondria by linking a specific cognitive construct (ie, cognitive fusion) with behavioral aspects (ie, excessive online health information seeking).

However, this study has several limitations that warrant consideration. First, our web-based sampling method, despite using various quality assurance techniques, may have introduced selection bias. Individuals unfamiliar with online surveys or those not part of the company’s panel may have been excluded, potentially limiting the generalizability of our findings. Second, although national census data for the population aged 60 years and older was unavailable, our sample was representative only to some extent of the demographic characteristics of China’s older population, as reported in previous studies. Specifically, our sample had higher proportions of educated individuals, men, and urban residents. These sampling differences may introduce bias in our findings. Third, our reliance on self-reported measures means that participants’ subjective views may have influenced their responses. Fourth, the study did not assess some potentially important variables, such as digital literacy. This was due to the limited length of the study questionnaire and the need to reduce the cognitive burden on older respondents. Variables like digital literacy may affect cyberchondria, and their direct or moderating effects should be explored in future studies. Fifth, our decision to model HRQOL using the EQ-5D-5L single utility score as a manifest variable in the SEM analysis deviates from conventional SEM practices. An alternative approach, such as conceptualizing HRQOL as a latent construct with the five EQ-5D-5L dimensions as formative indicators, might better capture the multidimensional nature of HRQOL. Although this alternative does not align with the standard use of the EQ-5D-5L utility score, it might reflect the differential contributions of each dimension and adhere more closely to typical SEM frameworks. Our approach may have constrained the model’s ability to account for the nuanced interplay of HRQOL dimensions, and future research should consider latent variable modeling to address this limitation. Last, our study used a cross-sectional design. Although SEM can offer some insights into potential causal relationships between variables, longitudinal data will be necessary in the future to establish definitive causal links.

### Conclusion

This study shows that almost two-thirds of the older Chinese online population might exhibit features of cyberchondria. Higher levels of cyberchondria were observed among older adults living in rural areas and those with lower socioeconomic status. Targeted educational programs could be useful for empowering older adults to better navigate cyberspace and identify credible online health information. Cyberchondria can enhance cognitive fusion in older populations, resulting in poorer HRQOL and mental well-being. Interventions aimed at “defusing” cyberchondria-relevant thoughts and breaking the cycle of anxiety-fueled, excessive online health information seeking can help reduce cyberchondria and improve the overall well-being of older populations.

## Appendix

Multimedia Appendix 1 The conceptual framework of the study and study hypotheses. Hypothesis 1 (H1): Cyberchondria correlates negatively with HRQoL and mental well-being in older adults. Hypothesis 2 (H2): Cognitive fusion mediates the relationships between cyberchondria and reduced HRQoL and mental well-being in older adults.

Multimedia Appendix 2 Survey questionnaire (English version).

Multimedia Appendix 3 Factor loadings of the SEM analysis.

## Abbreviations

CAPTCHA: Completely Automated Public Turing test to tell Computers and Humans Apart
CFQ: Cognitive Fusion Questionnaire
CSS-12: Cyberchondria Severity Scale-12
H1: hypothesis 1
H2: hypothesis 2
H3: hypothesis 3
H4: hypothesis 4
H5: hypothesis 5
SEM: structural equation modeling
WHO-5: World Health Organization-5 Well-Being Index

## Data Availability

Derived data supporting the findings of this study are available from the corresponding author on request.
