# Supplementary material for: Cyberchondria in Older Adults and Its Relationship With Cognitive Fusion, Health-Related Quality of Life, and Mental Well-Being: Mediation Analysis
Source: JMIR Aging. 2025 May 21;8:e70302. doi: 10.2196/70302 (PMC12138317; doi:10.2196/70302)
Supplement: Multimedia Appendix 1 [file aging_v8i1e70302_app1.docx]

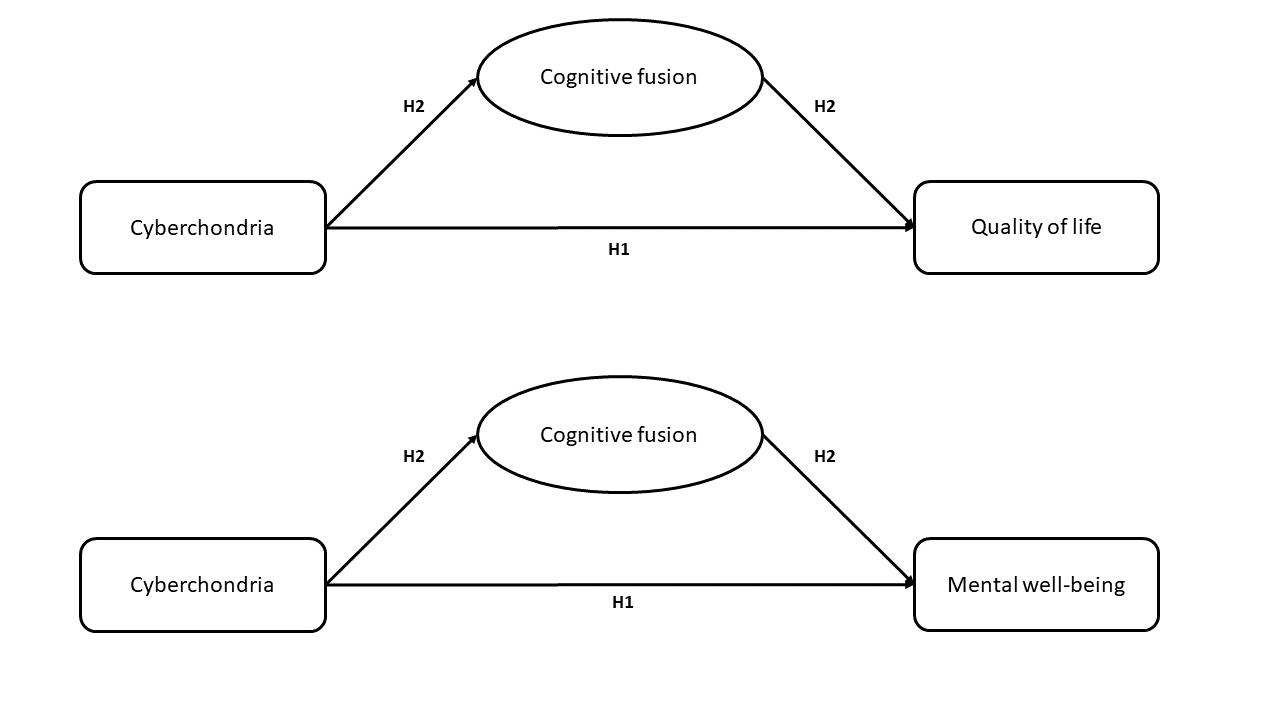


Figure 1. The conceptual framework of the study and study hypotheses. Hypothesis 1 (H1): Cyberchondria correlates negatively with HRQoL and mental well-being in older adults. Hypothesis 2 (H2): Cognitive fusion mediates the relationships between cyberchondria and reduced HRQoL and mental well-being in older adults.
