# Supplementary material for: Cyberchondria in Older Adults and Its Relationship With Cognitive Fusion, Health-Related Quality of Life, and Mental Well-Being: Mediation Analysis
Source: JMIR Aging. 2025 May 21;8:e70302. doi: 10.2196/70302 (PMC12138317; doi:10.2196/70302)
Supplement: Multimedia Appendix 2 [file aging_v8i1e70302_app2.docx]

**Research on cyberchondria, cognitive fusion, and quality of life**

We are conducting research on exploring the relationship among cyberchondria, cognitive fusion, and quality of life. We sincerely invite you to participate in this survey, and your opinions will provide an important basis for our research.

**Informed Consent Statement:**

This survey has been approved by the University Ethics Committee, and we are committed to keeping the data collected strictly confidential and using it for scientific research purposes only. **By submitting a survey, you are aware of all the contents of the survey and the purpose of the survey, and that you are willing to participate in the study.** During this process, you have the right to terminate your participation in this survey at any time.

**Part 1. Personal Information.**

| 1 Gender:  □1 male,  □2 female |
| --- |
| 2 Year of birth (e.g., 1970): ____________________ |
| 3Education level  □1 Elementary school and below  □2 Middle school (including junior high school and high school)  □ 3. University or above |
| 4. Household registration  □1. Urban household registration  □2. Rural household registration |
| 5. Do you live in your domicile  □1 Yes  □2 No |
| 6. Working conditions:  □1, full-time work,  □2, part-time work,  □3, retirement,  □4, unemployed,  □5, housewife |
| 7. Types of medical insurance:  □1 Urban employee insurance  □2. Urban resident insurance  □3 NCMS  □4. No insurance |
| 8 Marital Status  □ Single  □ Married  □ Divorced/Widowed |
| 9. Disposable annual household income:  □ 1 ≤ 50,000 □ 2 50,001~100,000  □ 3,100,001~200,000 □ 4,200,001~300,000  □ 5,300,001~400,000 □6 >,400,000 |
| 10 Have you been diagnosed with a chronic illness  □ Yes  □ No |

**Part 2. Cyberchondria (CSS-12).**

**Please read the following statements and indicate how they typically apply to you by circling the appropriate number. Please note that this questionnaire relates to perceived medical conditions (i.e., conditions you think you might have) rather than conditions that have been diagnosed by a medical profession.**

|  | never | seldom | sometimes | often | always |
| --- | --- | --- | --- | --- | --- |
| 1. If I notice an unexplained bodily sensation I will search for it on the internet |  |  |  |  |  |
| 1. Researching symptoms or perceived medical conditions online distracts me from reading news/ sports/ entertainment articles online |  |  |  |  |  |
| 1. I read different web pages about the same perceived condition |  |  |  |  |  |
| 1. I start to panic when I read online that a symptom I have is found in a rare/serious condition |  |  |  |  |  |
| 1. Researching symptoms or perceived medical conditions online leads me to consult with my GP |  |  |  |  |  |
| 1. I enter the same symptoms into a web search on more than one occasion |  |  |  |  |  |
| 1. Researching symptoms or perceived medical conditions online interrupts my work (e.g., writing emails, working on word documents or spreadsheets) |  |  |  |  |  |
| 1. I think I am fine until I read about a serious condition online |  |  |  |  |  |
| 1. I feel more anxious or distressed after researching symptoms or perceived medical conditions online |  |  |  |  |  |
| 1. Researching symptoms or perceived medical conditions online interrupts my offline social activities (e.g., reduces time spent with friends/family) |  |  |  |  |  |
| 1. I suggest to my GP/medical professional that I may need a diagnostic procedure that I read about online (e.g., a biopsy/a specific blood test) |  |  |  |  |  |
| 1. Researching symptoms or perceived medical conditions online leads me to consult with other medical specialists (e.g., consultants) |  |  |  |  |  |

**Part 3. Mental well-being (WHO-5).**

**Please respond to each item by marking one box per row, regarding how you felt in the last two weeks.**

|  | **All of the time** | **Most of the time** | **More than half the time** | **Less than half the time** | **Some of the time** | **At no time** |
| --- | --- | --- | --- | --- | --- | --- |
| I have felt cheerful in good spirits. | 5 | 4 | 3 | 2 | 1 | 0 |
| I have felt calm and relaxed. | 5 | 4 | 3 | 2 | 1 | 0 |
| I have felt active and vigorous. | 5 | 4 | 3 | 2 | 1 | 0 |
| I woke up feeling fresh and rested. | 5 | 4 | 3 | 2 | 1 | 0 |
| My daily life has been filled with things that interest me. | 5 | 4 | 3 | 2 | 1 | 0 |

**Part 4. Health-related quality of life (EQ-5D-5L).**

Under each heading, please tick the ONE box that best describes your health TODAY.

**MOBILITY**

1 I have no problems in walking about

2 I have slight problems in walking about

3 I have moderate problems in walking about

4 I have severe problems in walking about

5 I am unable to walk about

**SELF-CARE**

1 I have no problems washing or dressing myself

2 I have slight problems washing or dressing myself

3 I have moderate problems washing or dressing myself

4 I have severe problems washing or dressing myself

5 I am unable to wash or dress myself

**USUAL ACTIVITIES** *(e.g. work, study, housework, family or leisure activities)*

1 I have no problems doing my usual activities

2 I have slight problems doing my usual activities

3 I have moderate problems doing my usual activities

4 I have severe problems doing my usual activities

5 I am unable to do my usual activities

**PAIN / DISCOMFORT**

1 I have no pain or discomfort

2 I have slight pain or discomfort

3 I have moderate pain or discomfort

4 I have severe pain or discomfort

5 I have extreme pain or discomfort

**ANXIETY / DEPRESSION**

1 I am not anxious or depressed

2 I am slightly anxious or depressed

3 I am moderately anxious or depressed

4 I am severely anxious or depressed

5 I am extremely anxious or depressed

**Part 5. Cognitive fusion (CFQ).**

Please read the following statements carefully and select the option that best describes your situation.

|  |  | **Strongly Disagree** | **Disagree** | **Somewhat Disagree** | **Neutral** | **Somewhat Agree** | **Agree** | **Strongly Agree** |
| --- | --- | --- | --- | --- | --- | --- | --- | --- |
| 1 | Certain thoughts cause me distress and pain | 1 | 2 | 3 | 4 | 5 | 6 | 7 |
| 2 | I am so troubled by certain thoughts that I cannot complete tasks | 1 | 2 | 3 | 4 | 5 | 6 | 7 |
| 3 | I over-analyze certain situations, but it does not help me at all | 1 | 2 | 3 | 4 | 5 | 6 | 7 |
| 4 | I struggle with certain thoughts | 1 | 2 | 3 | 4 | 5 | 6 | 7 |
| 5 | I feel upset by certain thoughts | 1 | 2 | 3 | 4 | 5 | 6 | 7 |
| 6 | I need to control certain thoughts that appear in my mind | 1 | 2 | 3 | 4 | 5 | 6 | 7 |
| 7 | Certain thoughts trouble me deeply | 1 | 2 | 3 | 4 | 5 | 6 | 7 |
| 8 | I react strongly to certain thoughts | 1 | 2 | 3 | 4 | 5 | 6 | 7 |
| 9 | Although I know it's better to let go, I still get caught up in troubling thoughts | 1 | 2 | 3 | 4 | 5 | 6 | 7 |
