# Supplementary material for: Cyberchondria in Older Adults and Its Relationship With Cognitive Fusion, Health-Related Quality of Life, and Mental Well-Being: Mediation Analysis
Source: JMIR Aging. 2025 May 21;8:e70302. doi: 10.2196/70302 (PMC12138317; doi:10.2196/70302)
Supplement: Multimedia Appendix 3 [file aging_v8i1e70302_app3.docx]

Table a1 Factor loadings of the SEM analysis

| **latent variable** | **Item** | **Factor loading** | **SE** | **Z-score** | **P-value** | **ci.lower** | **ci.upper** |
| --- | --- | --- | --- | --- | --- | --- | --- |
| CYB | css1 | 0.643 | 0.025 | 25.836 | <0.001 | 0.595 | 0.692 |
|  | css2 | 0.616 | 0.029 | 21.498 | <0.001 | 0.559 | 0.672 |
|  | css3 | 0.636 | 0.028 | 22.883 | <0.001 | 0.582 | 0.691 |
|  | css4 | 0.682 | 0.025 | 26.935 | <0.001 | 0.633 | 0.732 |
|  | css5 | 0.551 | 0.033 | 16.526 | <0.001 | 0.486 | 0.617 |
|  | css6 | 0.687 | 0.023 | 29.863 | <0.001 | 0.642 | 0.733 |
|  | css7 | 0.62 | 0.029 | 21.644 | <0.001 | 0.563 | 0.676 |
|  | css8 | 0.638 | 0.025 | 25.169 | <0.001 | 0.588 | 0.687 |
|  | css9 | 0.632 | 0.025 | 25.039 | <0.001 | 0.582 | 0.681 |
|  | css10 | 0.538 | 0.03 | 17.775 | <0.001 | 0.479 | 0.597 |
|  | css11 | 0.476 | 0.034 | 14.184 | <0.001 | 0.411 | 0.542 |
|  | css12 | 0.523 | 0.034 | 15.396 | <0.001 | 0.457 | 0.59 |
| Well-being | who1 | 0.726 | 0.022 | 32.362 | <0.001 | 0.682 | 0.77 |
|  | who2 | 0.725 | 0.022 | 32.214 | <0.001 | 0.68 | 0.769 |
|  | who3 | 0.788 | 0.019 | 40.93 | <0.001 | 0.75 | 0.825 |
|  | who4 | 0.775 | 0.02 | 38.93 | <0.001 | 0.736 | 0.814 |
|  | who5 | 0.755 | 0.021 | 36.054 | <0.001 | 0.714 | 0.796 |
| Cognitive fusion | cog1 | 0.807 | 0.015 | 52.866 | <0.001 | 0.777 | 0.837 |
|  | cog2 | 0.804 | 0.015 | 51.959 | <0.001 | 0.773 | 0.834 |
|  | cog3 | 0.724 | 0.02 | 35.834 | <0.001 | 0.684 | 0.764 |
|  | cog4 | 0.831 | 0.014 | 60.408 | <0.001 | 0.804 | 0.858 |
|  | cog5 | 0.851 | 0.012 | 68.232 | <0.001 | 0.827 | 0.875 |
|  | cog6 | 0.774 | 0.017 | 44.824 | <0.001 | 0.74 | 0.808 |
|  | cog7 | 0.788 | 0.016 | 47.906 | <0.001 | 0.756 | 0.82 |
|  | cog8 | 0.773 | 0.017 | 44.611 | <0.001 | 0.739 | 0.807 |
|  | cog9 | 0.79 | 0.016 | 48.453 | <0.001 | 0.758 | 0.822 |
| HRQoL | MO | 0.778 | 0.027 | 28.965 | <0.001 | 0.725 | 0.831 |
|  | SC | 0.748 | 0.026 | 28.253 | <0.001 | 0.696 | 0.8 |
|  | UA | 0.794 | 0.024 | 32.811 | <0.001 | 0.747 | 0.842 |
|  | PD | 0.612 | 0.033 | 18.735 | <0.001 | 0.548 | 0.676 |
|  | AD | 0.586 | 0.033 | 17.987 | <0.001 | 0.523 | 0.65 |

CYB: Cyberchondria; HRQoL: Health-related quality of life.
